# Genomic Characterization of Clinical Canine Parvovirus Type 2c Infection in Wild Coyotes (*Canis latrans*) in Mexico

**DOI:** 10.3390/pathogens15010080

**Published:** 2026-01-11

**Authors:** Armando Busqueta-Medina, Ramiro Ávalos-Ramírez, Diana Elisa Zamora-Ávila, Víctor Eustorgio Aguirre-Arzola, Juan Francisco Contreras-Cordero, Sibilina Cedillo-Rosales

**Affiliations:** 1 Cuerpo Académico de Epidemiología Veterinaria, Facultad de Medicina Veterinaria y Zootecnia, Departamento de Virología, Universidad Autónoma de Nuevo León, Campus Ciencias Agropecuarias, Ave. Francisco Villa s/n, Ex-Hacienda el Canadá, General Mariano Escobedo C.P. 66054, Nuevo León, Mexico; armando.busquetamdn@uanl.edu.mx (A.B.-M.); diana.zamoravl@uanl.edu.mx (D.E.Z.-Á.); 2 Facultad de Agronomía, Universidad Autónoma de Nuevo León, Campus Ciencias Agropecuarias, Ave. Francisco Villa s/n, Ex-Hacienda el Canadá, General Mariano Escobedo C.P. 66054, Nuevo León, Mexico; victor.aguirrearz@uanl.edu.mx; 3 Laboratorio de Inmunología y Virología, Facultad de Ciencias Biológicas, Universidad Autónoma de Nuevo León, Ave. Universidad, S/N, Ciudad Universitaria, San Nicolás de los Garza C.P. 66450, Nuevo León, Mexico; juan.contrerascr@uanl.edu.mx

**Keywords:** *Protoparvovirus carnivoran* 1, CPV-2c, *NS1* mutation, *Canis latrans*, clinical gastroenteritis, northeastern Mexico

## Abstract

Canine parvovirus type 2 (CPV-2) is a primary etiological agent of acute gastroenteritis in domestic dogs. Although molecular and serological evidence have confirmed its circulation in wild carnivores, the clinical impact of spillover events in wildlife hosts remain insufficiently characterized. In this study, we investigated CPV-2 from wild coyote pups (*Canis latrans*) presenting with clinical gastroenteritis in northeastern Mexico. CPV-2 was successfully isolated in MDCK cells, and whole-genome sequencing was performed on two isolates, B55 and B56 (GenBank accession numbers PQ065988 and PQ065989). A comprehensive analysis identified 23 nucleotide mutations, eight of which were missense mutations resulting in amino acid substitutions in structural (VP) and non-structural (NS) proteins. Notably, amino acid substitution L354V was identified in the *NS1* helicase domain of both isolates, a region critical for viral replication. Phylogenetic analysis confirmed that isolates B55 and B56 cluster within the CPV-2c subtype, showing high genetic relatedness to circulating Mexican and US canine strains which strongly suggests recent cross-species transmission between domestic dogs and wild coyotes. This study provides the first complete genomic characterization of a clinical CPV-2 infection in wild coyotes in Mexico, underscoring the immediate risk of CPV-2c transmission at the domestic animal–wildlife interface.

## 1. Introduction

Canine parvovirus type 2 (CPV-2) is taxonomically classified as *Protoparvovirus carnivoran 1* within the family *Parvoviridae*, subfamily *Parvovirinae*, genus *Protoparvovirus* [1]. CPV-2 is a non-enveloped icosahedral virus with a negative single-stranded DNA (ssDNA), approximately 5 kb in length [2,3,4]. The CPV-2 genome contains two coding regions: at the 5′ end, ORF1 encodes the non-structural proteins NS1 and NS2, and at the 3′ end, ORF2 encodes the structural proteins VP1 and VP2 [5,6]. NS1 and NS2 proteins are responsible for viral replication and contribute to the virus’s cytotoxicity and pathogenicity [7,8]. VP1 and VP2 proteins assemble the viral capsid, with VP2 being the most abundant, comprising 90% of the capsid, and providing the virus with its antigenic properties by determining cell tropism and host range through interactions with the transferrin receptor (TfR) [9].

CPV-2 emerged in 1978 as a *Feline Panleukopenia Virus* (FPV) variant that affects the canine species with mutations K80R, K93N, V103A, D232N, N564S and A568G in *VP2* gene [10]. In 1980, the first CPV-2 variant (CPV-2a) appeared, with amino acid changes M87L, I101T, A300G, D305Y and V555I in *VP2* [2]. Shortly after, the second variant (CPV-2b) emerged, differing from variant 2a by amino acid change N426D and I555V in *VP2*, and the most recent variant (CPV-2c) appeared in 2000 with amino acid change D426E in *VP2* [2,4].

CPV-2 primarily affects domestic canines, causing lethargy, anorexia, vomiting, diarrhea, and in severe cases, hemorrhagic diarrhea with a high mortality rate [11]. However, CPV-2 has also been described in populations of wild carnivores [12,13], domestic pigs in the United States [14], and a clinical form of CPV-2 in pangolins in China and Taiwan [15,16]. Furthermore, studies have reported a high seroprevalence of CPV-2 in coyotes in the United States and Mexico [17,18,19,20]. Despite this, only one case has been reported in captive juvenile coyotes with severe diarrhea and high mortality associated with co-infection of CPV-2 and *Canine coronavirus* (CCoV) [21], limiting the ability to assess potential risks to wild coyotes and other wildlife populations in regions where interfaces between domestic animals, and wildlife are increasingly common.

Given the limited data on symptomatic CPV-2 in wild North American canids, this study presents the isolation and full genome characterization of CPV-2 from wild coyote pups with clinical gastroenteritis, representing the first such report in Mexico. Considering the risk CPV-2 poses to endangered canids and its capacity for cross-species transmission, these findings highlight the importance of wildlife as indicators of emerging viral variants at the intersection of urban and natural ecosystems.

## 2. Materials and Methods

### 2.1. Clinical Samples and Processing

Fecal samples from three coyote pups (two males, one female; age 2–3 months) exhibiting apathy, fever, and diarrhea were collected at Mariano Escobedo International Airport, N.L., Mexico. Samples were obtained using rectal swabs by personnel from the airport’s Wildlife Management Program, operated by Grupo Aeroportuario Centro Norte (OMA), and subsequently transported to the Department of Virology, Faculty of Veterinary Medicine, Universidad Autónoma de Nuevo León, for processing. Samples tested positive for CPV-2 via CPV Ab Test Kit 3.0 from Bionote (Hwaseong-si, Gyeonggi-do, Republic of Korea). PCR tested negative for *Canine coronavirus* (CCoV), *Canine distemper virus* (CDV), *Canine bocavirus* (CBoV), and *Canine circovirus* (CCV) and positive for CPV-2. Samples were homogenized in physiological saline solution at a 1:10 dilution and centrifuged at 3000× *g* for 10 min. The resulting supernatant was filtered through a 0.22 µm polyethersulfone (PES) membrane filter from Millipore Sigma (Burlington, MA, USA) for viral isolation.

### 2.2. CPV-2 Isolation

Madin–Darby Canine Kidney (MDCK) cells (ATCC® CCL-34™) were cultured in 25 cm^2^ culture flasks. Cells were maintained in high-glucose Dulbecco’s Modified Eagle Medium (DMEM) with L-glutamine and sodium pyruvate from Gibco™ (Grand Island, NY, USA), supplemented with 5% certified fetal bovine serum (FBS) from Gibco^TM^ and 1% Antibiotic-Antimycotic (100×) (penicillin, streptomycin, and amphotericin B) from Gibco™. The cells were maintained in a Sanyo CO_2_ incubator model MCO-19AIC(UV) (Panasonic Healthcare Co., Ltd., Tokyo, Japan) at 37 °C with 5% CO_2_ and 95% humidity.

Viral isolation was performed in 24-well plates with MDCK cells at 60–70% confluency, maintained in 200 µL of DMEM with 1% Antibiotic-Antimycotic. Filtered supernatant from the samples was added in triplicate at volumes of 100 µL, 25 µL, and 5 µL, and the plates were incubated for one hour at 37°C. After adsorption, the inoculum was removed, and 400 µL of DMEM supplemented with 5% FBS and 1% Antibiotic/Antimycotic was added. The plates were incubated for 3 to 7 days and observed daily for the development of a cytopathic effect (CPE). A PCR of the supernatants was performed to confirm CPV-2 replication.

### 2.3. Whole Genome Amplification and Sequencing

To ensure complete coverage of the CPV-2 whole genome, including both ORFs and UTRs, four overlapping primer pairs were designed (Table 1) using CPV-2 reference sequences retrieved from the GenBank database (National Center for Biotechnology Information, Bethesda, MD, USA) with the Primer-BLAST tool (NCBI) [22], version 2.5.0. Primers were synthesized by Integrated DNA Technologies (Coralville, IA, USA). PCRs for all primer pairs were performed under the following conditions: an initial denaturation cycle at 94 °C for 5 min, followed by 40 cycles with three temperature steps, denaturation at 94 °C for 30 s, primer annealing at 60 °C for 30 s, and extension at 72 °C for 1 min, followed by a final extension cycle at 72 °C for 10 min and 4 °C for 1 min. The resulting fragments were purified using the E.Z.N.A. ^®^ Gel Extraction Kit (Omega Biotek, Norcross, GA, USA) and were sequenced by Psomagen (Rockville, MD, USA).

### 2.4. Phylogenetic Analysis

Editing and assembly of the obtained sequences were performed using the SeqBuilder Pro program from DNASTAR Lasergene v.15 software suite (DNASTAR, Inc., Madison, WI, USA). Sequences were analyzed and compared with CPV-2 sequences from wild carnivores and domestic dogs to assess host-associated mutations and cross-species potential transmission.

Phylogenetic analyses were conducted with the MegAlign Pro program from DNASTAR Lasergene v.15. A multiple sequence alignment was performed using the Clustal W algorithm [23]. Genetic distances were estimated using the Kimura 2-Parameter method [24]. A dendrogram was constructed using the Neighbor-Joining method [25], and statistical support was assessed with a Bootstrap analysis of 1000 replicates [26].

## 3. Results

### 3.1. Isolation of CPV-2

CPV-2 isolation from the coyote feces was positive in all three samples, which were designated as isolates B54, B55, and B56. CPE was observed in the cultures, with cells becoming rounded and starting to detach from the monolayer on day 4 post-inoculation. CPV-2 replication was confirmed by PCR of the supernatants.

### 3.2. Amplification of the Complete CPV-2 Genome

**Figure 1:**
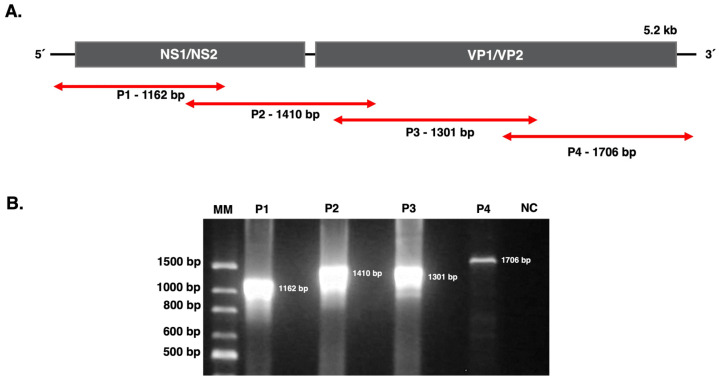
Whole genome amplification of CPV-2. (**A**) Schematic representation of CPV-2 genome and the four overlapping PCR fragments (P1–P4) used for whole-genome amplification. (**B**) Electrophoresis gel of the four overlapping amplicons spanning the entire CPV-2 genome from isolate B56, as shown in panel A. Expected amplicon sizes were approximately 1162 bp (P1), 1410 bp (P2), 1301 bp (P3), and 1706 bp (P4). M, molecular weight marker. NC, negative control (DEPC water).

Using the designed primers, four PCR products of 1162 bp, 1410 bp, 1301 bp, and 1706 bp were obtained from each of the CPV-2 isolates (Figure 1). Although full-genome amplification was achieved for isolate B54, this isolate was excluded from sequencing due to insufficient DNA concentration following gel purification. Two CPV-2 sequences of 4465 bp and 4469 bp were assembled for isolates B55 and B56 and submitted to GenBank with accession numbers PQ065988 and PQ065989, respectively. The assembled sequences include the complete coding regions of *NS1* (2007 nt, 669 aa), *NS2* (498 nt, 166 aa), *VP1* (2184 nt, 728 aa), and *VP2* (1755 nt, 585 aa) genes.

### 3.3. Sequence Analysis

**Figure 2:**
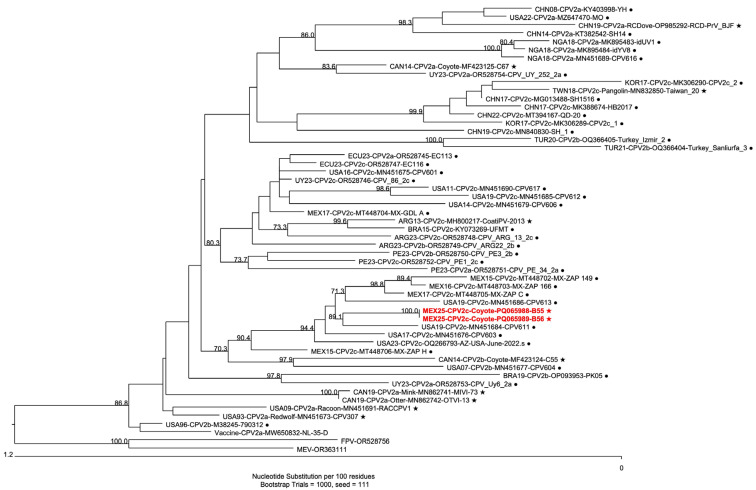
Phylogenetic tree based on complete genome sequences of CPV-2. Sequences derived from wildlife hosts are highlighted with ★, whereas dog-derived sequences are shown with •. Sequences from the present study (PQ065988 and PQ065989) are highlighted in red. Bootstrap values are displayed at the branching nodes. Only Bootstrap values ≥ 70 are shown.

A multiple alignment of complete sequences from CPV-2 strains isolated from different wildlife species and domestic dogs, along with the sequences from isolates B55 and B56 and the feline panleukopenia virus (FPV) and the mink enteritis virus (MEV) as outgroups, was performed. Sequences data are presented in Table S1. Isolates B55 and B56 (PQ065988 and PQ065989) share >98% of identity with the analyzed sequences. Based on the genetic distances, a phylogenetic tree was constructed (Figure 2), showing isolates B55 and B56 clustering within a clade together with other Mexican and United States sequences belonging to the CPV-2c subtype, with high bootstrap values.

**Figure 3:**
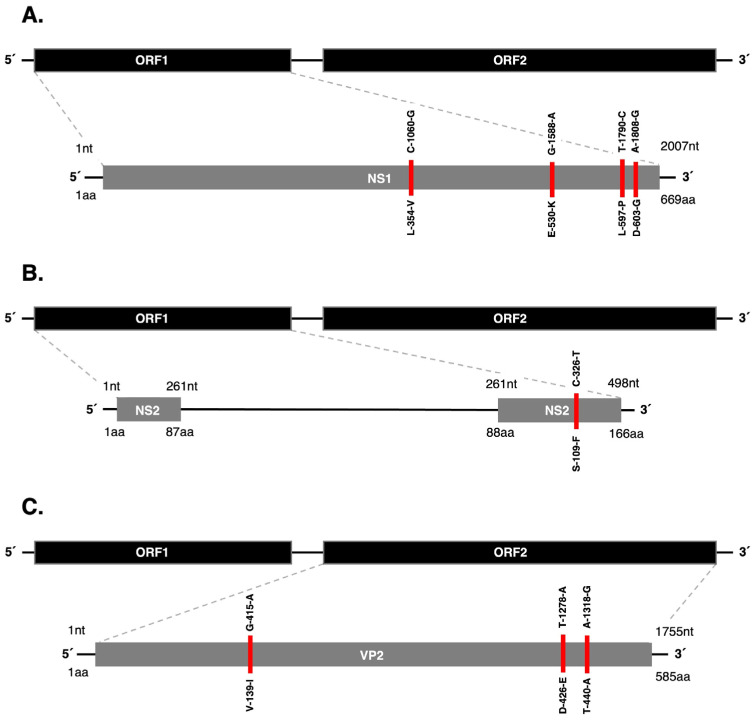
Genome organization of CPV-2 showing the mutations identified in isolates B55 and B56. The position of the mutation within the gene is highlighted in red. The nucleotide positions of the corresponding point mutations are shown above, while the resulting amino acid substitutions are indicated below. (**A**) Mutations in *NS1*. (**B**) Mutations in *NS2*. (**C**) Mutations in *VP2*.

Comparative analysis revealed that isolates B55 and B56 presented a total of 23 nucleotide mutations within the coding regions and no mutations in the untranslated regions (UTRs). Within ORF1, the *NS1* gene presented 9 mutations, 4 of which were missense mutations resulting in amino acid substitutions at residues L354V, E530K, L597P, and D603G (Figure 3A). In the *NS2* gene, 3 mutations were identified, one of which led to an amino acid substitution at residue S109F (Figure 3B). In ORF2, 11 mutations were detected, 3 of which were missense mutations corresponding to amino acid substitutions at residues V139I, D426E, and T440A in the *VP2* gene (Figure 3C). In Table 2 we summarize the amino acid substitutions detected in isolates B55 and B56 in comparison with the most closely related sequences, including wildlife-derived strains.

## 4. Discussion

Canine parvovirus type 2 (CPV-2) is a highly contagious virus that primarily affects domestic dogs, causing symptoms such as vomiting, lethargy, diarrhea, and it can be fatal, especially in young animals [11]. CPV-2 also affects wildlife, particularly carnivorous mammals CPV-2 [27]. In recent years, the virus has been reported on a pig farm in the United States [14], and clinical cases of CPV-2 have been documented in Asian pangolins [15,16], suggesting the emergence of new variants with a broader host range and increased pathogenicity.

Although several studies have documented the seroprevalence of CPV-2 in coyotes in North America [17,20,28,29], evidence of clinical disease in coyotes has been exceptionally scarce, limited to a single captive case [21]. In Mexico a seroprevalence of 86% was reported in feral dogs and other mammals [19] and more recently, the *VP2* gene was characterized in wild and domestic carnivores from a reserve in Mexico’s northwest, with a seroprevalence of 83% in coyotes [18]. In this study, CPV-2 was isolated and characterized from two coyote pups with clinical signs of gastroenteritis in northeastern Mexico. To our knowledge, this is the first report of a clinical form of CPV-2c infection in wild coyotes in Mexico.

Two complete CPV-2c sequences were established for isolates B55 and B56, which shared 100% nucleotide identity despite differences in sequence length. Although sequencing of isolate B54 could have provided further insight into the outbreak variability, B55/B56 homology supports the presence of a single, genetically stable viral lineage circulating during the outbreak. Additionally, isolates B55 and B56 showed > 98% of identity within the analyzed sequences. Phylogenetic analysis revealed that isolates B55 and B56 clustered with other Mexican and United States strains within a CPV-2c subclade, which is the predominant variant in Mexico [30,31]; however, CPV-2a has been reported in a fox from the northwest region of the country [18], and CPV-2b was identified in a dog with gastroenteritis form the northeastern region [32]. The low genetic divergence observed between isolates B55 and B56 and other Mexican strains suggests CPV-2 transmission between domestic dogs and wild coyotes, and potentially among wildlife in general, particularly other carnivores. Previous studies indicate that cross transmission of CPV-2 between domestic and wild animals frequently occurs in urban peripheries, where close contact between domestic carriers and wildlife facilitates viral spread [12,18,33].

Comparative analyses revealed that 52% of the mutations identified in isolates B55 and B56 were located in ORF1, which encodes the non-structural proteins NS1 and NS2, primarily responsible for viral replication [6,7,34,35]. Four amino acid substitutions were found in the *NS1* gene. Substitution L354V, located in the *NS1* helicase domain, was unique to isolates B55 and B56 within the analyzed dataset. Mutations within this domain may affect viral replication efficiency and, consequently, influence disease severity, as previous studies have demonstrated that *NS1* helicase domain plays a critical role in DNA binding and that mutations affecting this region may lead to reduced helicase activity, ultimately preventing the formation of infectious virions [34,35]. The E530K substitution was also detected in CPV-2 strains isolated from dogs in Mexico and the United States, whereas the L597P and D603G substitutions, located within the *NS1* transactivation domain, have been reported in CPV-2 strains from coyotes in Canada [13] and could potentially affect the regulation of capsid protein viral gene expression [35]. Other studies have described additional *NS1* mutations that were not detected in isolates B55 and B56 [36,37]. Mutations in *NS1* have been associated with reduced viral replication in CPV-2 [34,35,38], which may translate into decreased pathogenicity; however, *NS1* mutations in FPV have been shown to enhance viral replication [39]. In the *NS2* gene, a single amino acid substitution (S109F) was identified; this substitution has been previously reported in strains isolated from domestic dogs in Italy [8]. Although NS2 is not strictly essential for CPV-2 replication, it has been suggested to play a role in viral replication efficiency, intracellular trafficking, and interactions with host cell factors [6,7]. Even though the mutations identified in *NS1* and *NS2* could contribute to variations in viral replication, there is not enough evidence to conclude that they are determinant factors in the clinical presentation observed in the coyotes. Other host-related factors, such as immune response or environmental conditions, may also influence the severity of the disease. Therefore, targeted mutation studies are needed to clarify the specific role of these mutations in the pathogenesis of CPV-2 observed in the coyote pups.

On the other hand, 48% of the mutations were found in ORF2, which encodes the capsid proteins. CPV-2 binds to host cell TfR via VP2 protein; therefore, mutations in *VP2* play a key in determining the virus host range and have been associated with cross-species transmission events [40,41,42]. Three amino acid substitutions were identified in *VP2*. Substitution V139I is located between domains 1 and 2, which together form the threefold spike of CPV-2 capsid [43,44]. Notably, substitution V139I was observed only in isolates B55 and B56 among the sequences included in the comparative dataset. Further surveillance studies are needed to determine whether V139I represents a sporadic mutation or reflects broader circulation within coyote populations. Substitution T440A is located at the tip of the threefold spike, a key antigenic region of the capsid [45]. This mutation has been shown to reduce the binding energy between VP2 monomers [46], contributing to increased capsid stability, which could improve the virus environmental persistence and facilitate indirect transmission. Additionally, strains with substitution T440A tends to have lower antigenicity scores, suggesting that viruses carrying this substitution may evade host immune responses and exhibit enhanced immune resistance, driving the emergence of new CPV-2 variants [1,36,46,47].

These findings highlight the epidemiological relevance of wild canids in the maintenance and dissemination of CPV-2. Given the high mutation rate of approximately 10^−4^ substitutions per site per year, like RNA viruses [4,45], our results underscore the importance of implementing a One Health-based surveillance framework. Coyotes inhabiting the urban–wildlife interface may function both as sentinel species and as potential contributors to CPV-2 variant circulation, posing a tangible risk to the health of domestic dogs as well as to susceptible and endangered wild carnivore populations.

## Figures and Tables

**Table 1:** Primers designed to amplify the complete genome of CPV-2.

Name	Sequence (5′-3′)	*Tm* (°C)	Product Size (bp)	Nt. Position	GenBank Reference
P1-FWD	ACGGCAGTCACACGTCATAC	60.39	1162	72–91	M38245
P1-REV	CACCTCCTGGTTGTGCCATC	60.96		1233–1214	M38245
P2-FWD	AAACCACAGTGACGACAGCA	60.11	1410	1060–1079	M38245
P2-REV	CGGCGTCAGAAGGGTTAGTT	60.04		2469–2450	M38245
P3-FWD	CACAAAGACGTGCAAGCGAG	60.38	1301	2172–2191	M38245
P3-REV	GGTGTGCCACTAGTTCCAGTATG	60.93		3472–4350	M38245
P4-FWD	ATCTGCTACTCAGCCACCAAC	60.07	1706	3248–3268	MT448702
P4-REV	TTGATTAAGCAGAGCAACCAACC	59.74		4953–4931	MT448702

**Table 2:** Amino acid substitutions.

GenBank Reference	Subtype	Host	Country	AA Change (*NS1*)				AA Change (*NS2*)	AA Change (*VP2*)		
				L-354	E-530	L-597	D-603	S-109	V-139	D-426	T-440
MH800217	2c	Coati	Argentina	L	E	L	D	S	V	E	T
MF423125	2a	Coyote	Canada	L	E	L	D	S	V	N	T
MF423124	2b	Coyote	Canada	L	E	P	G	S	V	D	T
MN862741	2a	Mink	Canada	L	E	L	D	S	V	N	T
MN862742	2a	Otter	Canada	L	E	L	D	S	V	N	T
MT448702	2c	Dog	Mexico	L	K	P	G	S	V	E	A
MT448703	2c	Dog	Mexico	L	K	P	G	S	V	E	A
MT448705	2c	Dog	Mexico	L	K	P	G	S	V	E	A
MT448706	2c	Dog	Mexico	L	K	P	G	S	V	E	T
**PQ065988**	**2c**	**Coyote**	**Mexico**	**V**	**K**	**P**	**G**	**F**	**I**	**E**	**A**
**PQ065989**	**2c**	**Coyote**	**Mexico**	**V**	**K**	**P**	**G**	**F**	**I**	**E**	**A**
MN832850	2c	Pangolin	Taiwan	L	E	L	D	S	V	E	T
MN451691	2a	Raccoon	USA	L	E	L	D	S	V	N	T
OQ266793	2c	Dog	USA	I	K	P	G	S	V	E	A
MN451676	2c	Dog	USA	L	K	P	G	S	V	E	A
MN451684	2c	Dog	USA	L	K	P	G	S	V	E	A
MN451686	2c	Dog	USA	L	K	P	G	S	V	E	A
MN451673	2a	Red wolf	USA	L	E	L	D	S	V	N	T

Rows in bold correspond to isolates B55 and B56.

## Data Availability

All relevant data are provided in the present manuscript. Obtained sequences have been submitted to GenBank.Gen.

## Supplementary Materials

The following supporting information can be downloaded at: https://www.mdpi.com/article/10.3390/pathogens15010080/s1. Table S1: CPV-2 whole genome sequences data used in this study.

## Abbreviations

The following abbreviations are used in this manuscript:

A	Alanine
AA	Amino acid
Bp	Base pair
°C	Celsius
CBoV	*Canine bocavirus*
CDV	*Canine distemper virus*
CCV	*Canine circovirus*
CCoV	*Canine coronavirus*
cm	Centimeter
CO_2_	Carbon dioxide
CPE	Cytopathic effect
CPV-2	*Canine parvovirus type* 2
D	Aspartic acid
DEPC	Diethyl pyrocarbonate
DMEM	Dulbecco’s Modified Eagle Medium
DNA	Deoxyribonucleic acid
E	Glutamic acid
F	Phenylalanine
FBS	Fetal Bovine Serum
FPV	*Feline Panleukopenia Virus*
FWD	Forward
g	g-force unit
G	Glycine
I	Isoleucine
K	Lysine
kb	Kilo base
L	Leucine
LD	Linear dichroism
µm	Micron
µl	Microliter
M	Methionine
MDCK	Madin–Darby Canine Kidney
MEV	*Mink enteritis virus*
MDPI	Multidisciplinary Digital Publishing Institute
min	Minutes
N	Asparagine
NS	Non-structural protein
Nt	Nucleotide
ORF	Open Reading Frame
P	Proline
PCR	Polymerase Chain Reaction
R	Arginine
REV	Reverse
RNA	Ribonucleic acid
s	Seconds
S	Serine
ssDNA	Single-stranded DNA
T	Threonine
TfR	Transferrin Receptor
TLA	Three letter acronym
*Tm*	Melting temperature
UTR	Untranslated region
V	Valine
VP	Structural protein
Y	Tyrosine
