# Supplementary material for: Genomic Characterization of Clinical Canine Parvovirus Type 2c Infection in Wild Coyotes (Canis latrans) in Mexico
Source: Pathogens. 2026 Jan 11;15(1):80. doi: 10.3390/pathogens15010080 (PMC12845139; doi:10.3390/pathogens15010080)
Supplement: Supplementary file 1 [file pathogens-15-00080-s001.zip › pathogens-4083591-supplementary.pdf]

Table S1: CPV-2 whole genome sequences data used in this study.

No.	GenBank reference	Isolate/Strain	Genotype	Host	Country
1.	MH800217	CoatiPV-2013	CPV-2c	Coati	Argentina
2.	OR528749	CPV_ARG22_2b	CPV-2b	Canine	Argentina
3.	OR528748	CPV_ARG_13_2c	CPV-2c	Canine	Argentina
4.	KY073269	UFMT	CPV-2c	Canine	Brazil
5.	OP093953	PK05	CPV-2b	Canine	Brazil
6.	MF423125	C67	CPV-2a	Coyote	Canada
7.	MF423124	C55	CPV-2b	Coyote	Canada
8.	MN862741	MIVI-73	CPV-2a	Mink	Canada
9.	MN862742	OTVI-13	CPV-2a	Otter	Canada
10.	KY403998	YH	CPV-2a	Canine	China
11.	KT382542	SH14	CPV-2a	Canine	China
12.	MG013488	SH1516	CPV-2c	Canine	China
13.	MK388674	HB2017	CPV-2c	Canine	China
14.	OP985292	RCD-PrV_BJF6	CPV-2a	Dove	China
15.	MN840830	SH_1	CPV-2c	Canine	China
16.	MT394167	QD-20	CPV-2c	Canine	China
17.	OR528745	EC113	CPV-2a	Canine	Ecuador
18.	OR528747	EC116	CPV-2c	Canine	Ecuador
19.	OR528756	FPV_9_AR	FPV	Cat	Argentina
20.	MK306289	CPV2c_1	CPV-2c	Canine	Korea
21.	MK306290	CPV-2c_2	CPV-2c	Canine	Korea
22.	OR363111	MEV_SD-4	MEV	Mink	China
23.	MT448702	MX-ZAP 149	CPV-2c	Canine	Mexico
24.	MT448706	MX-ZAP H	CPV-2c	Canine	Mexico
25.	MT448703	MX-ZAP 166	CPV-2c	Canine	Mexico
26.	MT448704	MX-GDL A	CPV-2c	Canine	Mexico
27.	MT448705	MX-ZAP C	CPV-2c	Canine	Mexico
28.	MK895483	idUV1	CPV-2a	Canine	Nigeria
29.	MK895484	idYV8	CPV-2a	Canine	Nigeria
30.	MN451689	CPV616	CPV-2a	Canine	Nigeria
31.	OR528751	CPV_PE_34_2a	CPV-2a	Canine	Peru
32.	OR528750	CPV_PE3_2b	CPV-2b	Canine	Peru
33.	OR528752	CPV_PE1_2c	CPV-2c	Canine	Peru
34.	OQ366405	Turkey_Izmir_2	CPV-2b	Canine	Turkey
35.	OQ366404	Turkey_Sanliurfa_3	CPV-2b	Canine	Turkey
36.	MN832850	Taiwan_2018	CPV-2c	Pangolin	Taiwan

37.	MN451677	CPV604	CPV-2b	Canine	USA
38.	MN451691	RACCPV1	CPV-2a	Raccoon	USA
39.	MN451690	CPV617	CPV-2c	Canine	USA
40.	MN451679	CPV606	CPV-2c	Canine	USA
41.	MN451675	CPV601	CPV-2c	Canine	USA
42.	MN451676	CPV603	CPV-2c	Canine	USA
43.	MN451686	CPV613	CPV-2c	Canine	USA
44.	MN451684	CPV611	CPV-2c	Canine	USA
45.	MN451685	CPV612	CPV-2c	Canine	USA
46.	MZ647470	MO	CPV-2a	Canine	USA
47.	OQ266793	AZ-USA-June-2022	CPV-2c	Canine	USA
48.	MN451673	CPV307	CPV-2a	Red wolf	USA
49.	M38245	790312	CPV-2b	Canine	USA
50.	OR528753	CPV_Uy6_2a	CPV-2a	Canine	Uruguay
51.	OR528754	CPV_UY_252_2a	CPV-2a	Canine	Uruguay
52.	OR528746	CPV_86_2c	CPV-2c	Canine	Uruguay
53.	MW650832	NL-35-D	CPV-2	Vaccine	China
54.	PQ065988	B55	CPV-2c	Coyote	Mexico
55.	PQ065989	B56	CPV-2c	Coyote	Mexico
